# Complete Genome Sequences of *Lactococcus lactis* subsp. *lactis* bv. diacetylactis FM03 and *Leuconostoc mesenteroides* FM06 Isolated from Cheese

**DOI:** 10.1128/genomeA.00633-17

**Published:** 2017-07-13

**Authors:** Oscar van Mastrigt, Tjakko Abee, Eddy J. Smid

**Affiliations:** Laboratory of Food Microbiology, Wageningen University & Research, Wageningen Campus, Wageningen, The Netherlands

## Abstract

Here, the genome sequences of *Lactococcus lactis* subsp. *lactis* bv. diacetylactis FM03 and *Leuconostoc mesenteroides* FM06, both isolated from cheese, are presented. FM03 and FM06 contain 7 and 3 plasmids, respectively, that carry genes encoding functions important for growth and survival in dairy fermentations.

## GENOME ANNOUNCEMENT

*Leuconostoc mesenteroides* and *Lactococcus lactis* subsp. *lactis* bv. diacetylactis are two species of lactic acid bacteria present in starter cultures for cheese production. Compared to *L. lactis* subsp. *cremoris* strains, *L. mesenteroides* and *L. lactis* subsp. *lactis* strains show better survival during cheese ripening and thus provide an important contribution to the flavor development (1). Moreover, these two species of lactic acid bacteria also have the ability to utilize citrate, enhancing the production of the buttery aromas acetoin and diacetyl (2). In *L. lactis* subsp. *lactis*, the gene for citrate transport is located on a plasmid (3), showing the importance of plasmids for lactococcal performance in dairy applications. Another trait found regularly encoded on plasmids is the resistance to bacteriophages (4).

*Lactococcus lactis* subsp. *lactis* bv. diacetylactis FM03 and *Leuconostoc mesenteroides* FM06 were isolated from 10-week-old Samsø cheese. Their genomes were sequenced using an Illumina HiSeq 2500 and a PacBio RS instrument. Respectively, 6.4 and 4.3 million quality-filtered paired-end Illumina sequence reads of 100 bp were *de novo* assembled into contig sequences using CLC Genomics Workbench version 7.0.4 (CLC bio, Aarhus, Denmark). The contigs were linked and placed into scaffolds based on alignment of the PacBio reads (total, 167 Mbp and 493 Mbp, respectively). This resulted in 8 and 4 scaffolds for FM03 and FM06, respectively. Three out of 12 scaffolds were already circular. The remaining scaffolds were closed with PCR, followed by Sanger sequencing. The complete sequence of *L. lactis* FM03 was annotated using RAST (5) and manually curated. The sequence of *L. mesenteroides* FM06 was annotated with the NCBI Prokaryotic Genome Annotation Pipeline (6).

The genome sequence of *L. lactis* FM03 contains a chromosome of 2.43 Mbp, with a G+C content of 35.3%, and 7 plasmids with sizes of 3.4, 4.2, 7.5, 8.3, 12.0, 15.2, and 30.3 kbp and G+C contents of 33.8, 35.6, 33.6, 34.8, 33.5, 34.1, and 35.2%, respectively. The plasmids contained genes involved in citrate utilization (*citQRP*), resistance to bacteriophages (five different specificity subunits of a type I restriction-modification system), and resistance to several stresses. In most plasmids, transposons were also identified.

The genome sequence of *Leuconostoc mesenteroides* FM06 contains a chromosomal sequence of 1.89 Mbp, with a G+C content of 35.3%, and 3 plasmid sequences with sizes of 8.8, 15.3, and 31.1 kbp and G+C contents of 35.2, 39.2, and 36.0%, respectively. The plasmids contained genes involved in metal transport, lactose utilization, and resistance to bacteriophages. Interestingly, genes for lactose utilization (lactose phosphotransferase system [PTS] encoded by *lacS* [7] and β-galactosidase encoded by *lacLM* [8]) were carried by two different plasmids.

Further investigations into the genomes of these lactic acid bacteria may provide more insight into the role of plasmids in growth and survival in dairy fermentations.

### Accession number(s).

The genome sequences of *Lactococcus lactis* FM03 and *Leuconostoc mesenteroides* FM06 have been deposited at GenBank under the accession numbers CP020604 to CP020611 and CP020731 to CP020734, respectively.
